# Multiscale Determinants Drive Parasitization of Drosophilidae by Hymenopteran Parasitoids in Agricultural Landscapes

**DOI:** 10.3390/insects11060334

**Published:** 2020-05-30

**Authors:** Valeria Trivellone, Michela Meier, Corrado Cara, Lucia Pollini Paltrinieri, Felix Gugerli, Marco Moretti, Sarah Wolf, Jana Collatz

**Affiliations:** 1 WSL Swiss Federal Research Institute, Zürcherstrasse 111, 8903 Birmensdorf, Switzerland; michela.meier31@gmail.com (M.M.); felix.gugerli@wsl.ch (F.G.); marco.moretti@wsl.ch (M.M.); 2 Illinois Natural History Survey, University of Illinois at Urbana-Champaign, Champaign, IL 61821, USA; corradoc@illinois.edu; 3 Museo Cantonale di Storia Naturale, Viale C. Cattaneo 4, 6900 Lugano, Switzerland; lucia.pollini@ti.ch; 4 Agroscope, Research Division Agroecology and Environment, Agroscope, Reckenholzstrasse 191, 8046 Zurich, Switzerland; sarah.wolf@agroscope.admin.ch (S.W.); jana.collatz@agroscope.admin.ch (J.C.)

**Keywords:** parasitoid community, environmental driver, drosophilid

## Abstract

(1) The management of agricultural landscapes for pest suppression requires a thorough understanding of multiple determinants controlling their presence. We investigated the ecological preferences of indigenous parasitoids and their drosophilid hosts to understand the role of native parasitoids as biological control agents of the invasive frugivorous *Drosophila suzukii*. (2) Using data from an extensive field survey across different habitat types we analyzed the influence of abiotic and biotic factors on parasitoid and drosophilid communities at multiscale levels. (3) Eight parasitoid and 27 drosophilid species were identified. Thirty-four percent variation in drosophilid communities was explained by factors at the landscape scale, and 52% of significant variation of parasitoids by local distribution of three drosophilid species, mainly collected in woodland. Parasitoid communities were significantly influenced by microhabitat type (ground versus canopy) rather than habitat type. All parasitoids except *Pachycrepoideus vindemmiae* preferred the ground microhabitat. All parasitoids, with the exception of *Trichopria drosophilae* and *Spalangia erythromera*, displayed significant preferences among the drosophilid species used in the baited traps. (4) Since they can tolerate a broad range of habitat factors, altogether pupal parasitoids investigated in this study could play a role in biological control programs to suppress *D. suzukii*, but non-target effects have to be regarded.

## 1. Introduction

Parasitoids, i.e., insects that develop in or on another arthropod thereby causing its death, are among the most important organisms in biological control [1]. Host–parasitoid associations are structured by genetic and physiological interactions among the species that are ultimately influenced by environmental conditions [2,3]. It has been shown that environment-mediated effects on host–parasitoid interactions strongly affect species competition within and among trophic levels [4]. To survive and thrive, parasitoids need various resources such as hosts, alternative food sources, suitable microclimate, and refuges [5]. In the crop environment those needs may not be fulfilled in all locations and at all times and thus the presence of semi-natural (SNH) and remnant habitats (RH, undisturbed natural area) may enhance pest control [6,7]. For example, alternative hosts may sustain parasitoid populations outside of the cropping season or sheltered conditions are necessary for overwintering [8]. Higher persistence and fitness of parasitoids has been shown when woodlands and permanent grasslands were present in the vicinity of crops (e.g., [9,10]). However, polyphagous herbivores including pest species can also profit from SNHs and RHs because they likewise find alternative food sources and shelter when conditions in the crop are not optimal (e.g., [11]). The deliberate management of the agricultural landscape to suppress pests therefore requires a thorough understanding of the interplay among determinants for pest and parasitoid presence.

A species that has recently emerged as an important agricultural pest causing large economic damages to fruit production, is the frugivorous spotted wing drosophila *Drosophila suzukii* (Matsumura, 1931) (Diptera: Drosophilidae) [12,13]. The species is endemic to Asia but has now invaded Europe, and the Americas [14,15]. Its development inside the fruit, its polyphagous nature with more than 100 fruit species known so far to support larval development [16,17,18], and the high mobility of adults [19] make this species difficult to control. While the use of insecticides can deliver short-term control [20], re-infestation from the surroundings, i.e., neighboring farms or adjacent SNHs or RHs, may occur fast. Indeed, *D. suzukii* has been found to appear earlier in crops adjacent to forests [21,22] and to early season hosts [23]. In some studies [24,25], the overall densities of *D. suzukii* were correlated with forest cover, thus they also appear to profit from the presence of SNHs and RHs.

Numerous indigenous parasitoids attacking the larvae and pupae of various species of Drosophilidae are found worldwide, most of them are generalist to a certain extent, while no parasitoids of eggs or adults are known [26]. When *D. suzukii* became established in Europe and North America, several studies characterized indigenous parasitoids that could potentially include *D. suzukii* in their host spectrum [27,28,29,30,31]. In Europe, hymenopteran larval parasitoids, *Asobara tabida* (Nees, 1834) (Braconidae), *Leptopilina boulardi* (Barbotin, Carton and Kelner-Pillault, 1979) and *L. heterotoma* (Thomson, 1862) (Figitidae), did either not parasitize *D. suzukii* or their eggs were encapsulated by the host [32]; however, egg laying by the latter two still reduced the fitness of *D. suzukii* [30,33]. In contrast, the pupal parasitoids *Trichopria drosophilae* (Perkins, 1910) (Diapriidae), *Pachycrepoideus vindemmiae* (Rodani, 1875), *Spalangia erythromera* Forster, 1850 and *Vrestovia fidenas* (Walker, 1848) (all: Pteromalidae) were able to successfully parasitize *D. suzukii* [30,32,34].

Previous studies showed that the geographical distribution of parasitoids of drosophilids is affected by environmental factors such as temperature [35,36,37]. Since larval and pupal parasitoids exhibit various degrees of polyphagy, their association with drosophilid species may be deeply driven by the interaction of multiple biotic and abiotic factors acting at different spatial scales. Kremmer et al. [31] pointed out that some native species of drosophilids showed similar ecological preferences to *D. suzukii* and thus could be affected as non-target species in biological control programs because they could be included in the range of hosts from the suite of natural enemies. A recent study focusing on *D. suzukii* showed a positive relationship between forest cover and landscape complexity within a 1.5 km radius and the presence and abundance of parasitoids [24], yet only a minor proportion of those parasitoids belonged to species that parasitize *Drosophila*. Few studies investigated how parasitoids, which can attack *D. suzukii*, are influenced by alternative hosts in the field (e.g., [38]), and the effect of multiscale factors affecting the entire communities of drosophilids and their parasitoids remain poorly understood [39].

Our study aims to investigate the ecological preferences of the indigenous parasitoids of drosophilids and the interaction between abiotic and biotic factors in affecting both parasitoid and drosophilid communities in different habitat types at different spatial scales in Switzerland. In particular, we assessed (i) the influence of landscape- versus local level variables on the community composition of parasitoids and drosophilids, (ii) the influence of habitat and microhabitat types on the community structure of parasitoids and abundance of single parasitoid species, and (iii) the host preference of parasitoid species inside traps exposed in the field.

## 2. Materials and Methods

### 2.1. Study Site

The study was carried out in two biogeographic regions in Switzerland, the Canton of Ticino (hereafter Ticino) situated south of the Swiss Alps and the Canton of Zurich (Zurich) in the Northeast of Switzerland. The importance of habitat and microhabitat factors was investigated in both regions, and an extended sampling was only conducted in Ticino because the agricultural landscape complexity makes it a suitable study region to test the multiscale determinants (landscape- and local-scales) affecting drosophilid and parasitoid communities. In Ticino, half of the land is covered by forest and the agricultural landscapes (including mainly orchards, vineyards, greenhouses and gardens) is highly fragmented. The insubric climate of the study region is influenced by the presence of lakes and alpine ranges, which define the climatic conditions characterized by winters that are normally dry and sunny, sometimes windy (Foehn from the North), and with periods of snow accumulation, and warm, often rainy summers. The mean annual precipitation ranges from 1600 (S) to 1700 mm (N), and mean monthly temperatures from 0.5 (N) to 1.6 °C (S) in January and from 21.2 (N) to 23.5 °C (S) in July [40]. To cover the large landscape variability across this region 16 sites were selected, eight localities in rather homogeneous landscapes with semi-natural woodland and only one main agroecosystem type (vineyards, berries or orchards) and eight localities in heterogeneous landscapes (Table 1). Heterogeneous landscapes contained more than one crop in an area of at least 30,000 m^2^, with mixtures of semi-natural woodland and different kinds of small, scattered agroecosystems (mainly vineyards, blackberries, raspberries, cherry trees). The Canton of Zurich is the most densely populated area in Switzerland with about 40% agricultural land (of those about 50% arable land, 44% meadows and relatively small proportions of 0.8% and 0.5% vineyards and intensive orchards, respectively), 30% forest and 20% settlement area [41]. Mean annual precipitation in Zurich is 1100 mm and mean monthly temperatures are −0.6 °C in January and 17.6 °C in July [42].

### 2.2. Biological Sampling and Explanatory Variables

Stock cultures of four native drosophilid species (*Drosophila hydei* Sturtevant, 1921 (DH), *D. immigrans* Sturtevant, 1921 (DI), *D. melanogaster* Meigen, 1830 (DM), *D. subobscura* Collin, 1936 (DO)) were reared on an artificial diet (400 g banana, 20 g agar-agar, 50 g brewer’s yeast, 30 g wheat flour, 20 g saccharose, 4 g methylparaben, 1 l water). Adult flies were kept in flight cages that contained blocks of the artificial diet for food and oviposition. The diet was replaced every other day and the diet containing *Drosophila* spp. eggs was kept in ventilated plastic jars until emergence of adult flies. For sample preparation, plastic cups were filled with ripe seasonal fruit (e.g., cherry, plum) that were pierced multiple times with a needle to give the flies access for oviposition. The samples were exposed in the flight cages for 48 h and were then stored for 5 d at 22°C until formation of the first *Drosophila* spp. pupae. Artificial diet infested with the respective *Drosophila* species of similar age was added to the fruit samples before exposure in the field.

A multiscale approach was used to assess the potential drivers (abiotic and biotic) of parasitoids and drosophilids communities (Spatial scale, Table 2). In Ticino, environmental variables were measured at landscape and local scale. At landscape scale, four variables were included: the proportion of all crop land (*crop_500*), proportion of woody land (*woody_500*), patch density (PD) of crop (*PD_crop_500)* and PD of woody land (*PD_woody_500*). For each locality, the first two variables were measured in m^2^ digitizing the area within a radius of 500 m using the geoportal geo.admin.ch (https://map.geo.admin.ch). PD was calculated as the number of patches per unit area (100 hectares). Low PD indicates a low level of landscape fragmentation [43]. Local level variables included detailed information about the potential plant hosts of Drosophilidae (proportion of vineyard land- *vineyard_100*, proportion of cultivated berries land- *berries_100*, presence of berries in the wild- *berries_wild* and presence of fruit trees in the wild- *tree_wild*) and abundance of drosophilids (see below). The proportion of vineyard and berries land were digitized as above within a radius of 100 m surrounding the handmade modified Delta-traps (see description in Figure S1) allocated to collect parasitoids. The presence of single wild trees and berries were recorded in the field and coded as a categorical variable according to a scale of preference as host of Drosophilidae of 1 or 2 following results from the recent literature [16,17,44] (Table S1). In Ticino, adults of Drosophilidae were sampled in the field at the same time as the parasitoids, using Gasser-Becherfalle traps (Organic fruit fly traps for *Drosophila suzukii*, Riga AG, Switzerland) baited with a wine-vinegar-based attractant and covered by a white lid. At each locality, two traps were placed, one in the crop and one in the semi-natural/woody habitat; each trap at a distance of approx. 5 m from the Delta-traps. A total of 160 Gasser samples were collected (16 sites × 5 periods × 2 habitats (crop and woody)).

In Ticino, three different habitats were sampled in each locality: crop (crop), semi-natural or natural woodland (woody), and ecotone area between woodland and crop (ecotone). Only crop and woody habitats were inspected in Zurich. For each habitat type, two microhabitat types were distinguished: ground level (5 cm above ground) and vegetated canopy level (1–1.5 m above ground). To collect parasitoids and test their host preference in the field (within Delta-trap, Table 2), Delta-traps were baited with fruit containing larvae and pupae of the four native drosophilid species that were allocated in four separate plastic cups (Figure S1). A total of six Delta-traps were placed at each locality, two in each habitat type, one at ground and one at canopy level at a minimum distance of 5 m from each other to avoid interferences. The trap at ground level was protected with a poultry netting to avoid damage by various mammals or big snails.

The Delta-traps were exposed in the field for about 4–5 days during five periods in 2017, i.e., May (only semi-natural habitat), June, August, September, and October. The traps were moved in space and time according to the maturation period of the fruit in the field. A total of 416 Delta-traps (samples) in Ticino (16 sites × 4 periods (June, August, September, and October) × 3 habitats × 2 microhabitats + 16 sites × 1 period (May) × 1 habitat (woody) × 2 microhabitats) and 112 in Zurich (8 sites × 3 periods (June, July, and September) × 2 habitats × 2 microhabitats + 8 sites × 1 period (May) × 1 habitat (woody) × 2 microhabitats) were deployed.

### 2.3. Laboratory Work

Each sample collected from Delta-traps was kept separately in the laboratory at 23 °C and 70% (Zurich) or ambient relative humidity (average: 56%, Ticino) in emerging chambers for about 6 weeks. The emerging chambers in Ticino were humidified by spraying with water twice a week. After about 1–2 weeks non-parasitized *Drosophila* individuals emerged and were removed from the emerging chambers. Parasitoids started to emerge after about 3–4 weeks and were collected and preserved in 96% ethanol at −20 °C. Morphological characters of all parasitoid specimens were inspected under the stereoscope, thereafter identified by using published taxonomic keys and related literature [45,46,47,48,49,50].

The drosophilid specimens collected by each Gasser-Becherfalle trap were sorted and preserved in 96% ethanol. All adults were identified to species level by using Bächli et al. [51], and the individuals were tallied. Voucher specimens were deposited at the Natural History Museum in Lugano.

### 2.4. Data Analyses

Multivariate analyses were applied to highlight patterns of variability of the entire community of parasitoids (i.e., the abundance and list of species for each site). The overview of the analyses is reported in Table 2. Data of the extended sampling in Ticino were analyzed using redundancy analysis (RDA) to reveal the variation of parasitoid and drosophilid communities among localities that can be explained by landscape and local level variables (Table 2). First, for each spatial scale (landscape and local) and response variable (parasitoid and drosophilid communities), separate RDAs were computed to detect the effects of landscape and local level abiotic variables on parasitoids and drosophilid communities. Second, the species abundances of Drosophilidae were used as explanatory biotic variables to detect the effect of host on the variation in parasitoid species composition. When used as explanatory variables, species of drosophilids were included in the analysis after forward selection according to Dray et al. [52] (*p* = 0.05 after 9999 random permutations). All environmental variables were standardized, and correlated explanatory variables (>0.60) were discarded in each step. Abundance matrices of parasitoids and drosophilids were pooled for each sampling site, Hellinger-transformed and singleton (i.e., species with less than five individuals as total) removed [53]. The significance of canonical axis and of explanatory variables was evaluated by Monte Carlo permutation test using 1000 permutations (*p* > 0.05).

Data of parasitoids collected in Ticino and Zurich were analyzed using a distance-based permutational multivariate analysis of variance (PERMANOVA, [54]) to investigate variations of communities of parasitoids at habitat and microhabitat level (Table 2). Three variables (factors) were included in the analysis: habitat type (three levels: crop, ecotone, woody), microhabitat type (two levels: ground, canopy), and collecting period (five levels: from May to October). The site was considered to be a random factor. The analysis was applied to an untransformed abundance matrix of parasitoids (528 samples × 8 species) using Bray-Curtis dissimilarities [55]. Significance (*p* value) was calculated using a Monte Carlo test and residuals were permuted under a completely randomized model [56]. We tested the hypothesis of no significant difference in the species composition between/among groups (period, habitat and microhabitat). As the analysis is sensitive to the differences in the within-group dispersions, we used PERMDISP [57] to test for the homogeneity of dispersion among groups [58]. The effect of microhabitat was further analyzed for each parasitoid species separately using non-parametric Man-Whitney U tests for independent samples.

The effect of the four *Drosophila* species used as bait on the emergence of parasitoids from samples within the traps collected in Ticino and Zurich (host preference, Table 2) was analyzed using non-parametric Friedman 2-factor analysis of variance for dependent samples, followed by Wilcoxon signed rank test for dependent samples corrected with the false discovery rate method [59].

All statistical analyses were carried out using the software R [60] and packages ‘vegan’, ‘ade4’, and ‘rich’ or IBM SPSS 24.

## 3. Results

### 3.1. Faunistic Surveys

Overall, 8677 parasitoid individuals belonging to eight species emerged from 528 Delta-trap samples placed in Ticino and Zurich in 2017 (Table 3).

Among them, three species parasitize the larval stage of the host and five species the pupal stage. In Ticino, the four most abundant species were *T. drosophilae* (926 individuals), *L. boulardi* (649), *P. vindemmiae* (646), and *L. heterotoma* (426) whereas *L. heterotoma* was the most abundant and dominant species in Zurich (5316, Table 3). *Trichopria drosophilae* and *P. vindemmiae* were the most widespread species collected in Ticino (13 localities out of 16 sampled, Figure S2 and Table 3), and *T. drosophilae* was very rare in Zurich (collected in 1 site out of 8 sampled).

In Ticino, a total of 27 species and 20,947 individuals of Drosophilidae were recorded in 2017 (Table S2). The most abundant species were *D. melanogaster* (23% of individuals caught), *D. subobscura* (15%), *D. immigrans* (12%), *D. busckii* Cocquillett, 1901 and *D. kuntzei* Duda, 1924 (10%). The exotic *D. suzukii* represented only 3% of individuals. The most widespread species detected in all the sites were *D. hydei*, *D. immigrans*, *D. kuntzei*, *D. melanogaster*, *D. obscura* Fallen, 1823, *D. phalerata* Meigen, 1830, *D. simulans* Sturtevant, 1919, *D. subobscura*, *D. suzukii*, and *D. testacea* von Roser, 1840. Only one drosophilid species was almost exclusively collected inside the crop (*Scaptomyza pallida* (Zetterstedt, 1847)). Four species were almost equally collected inside the crop and woody habitat (*Chymomyza amoena* (Loew, 1862), *Scaptomyza graminum* (Fallen, 1823), *D. simulans*, and *D. melanogaster*). Most of the species showed a higher preference for woody habitat with a percentage of specimens collected in total ranging from 73% to 100%. Excluding *Amiota albilabris* (Roth, 1860), *D. littoralis* Meigen, 1830, and *Leucophenga maculata* (Dufour, 1839) that were collected with only very few individuals, the highest fidelity for woody land ranging from 99% to 96% of individuals was observed for *D. helvetica* Burla, 1948, *D. kuntzei*, *D. phalerata*, *D. testacea*, *D. immigrans*, *D. obscura*, and *D. funebris* (Fabricius, 1787) (Table S2).

### 3.2. Factors Affecting Parasitoids

#### 3.2.1. Influence of Landscape Versus Local Variables on Community of Parasitoids and Drosophilids

In Ticino, explanatory variables calculated for the 16 sites at landscape level (500 m radius) showed that on average 34% and 33% of the area was covered by crops and woody land, respectively. The average (±sd) of patch density for crop was 16.01 ± 8.16, whereas for woody land it was significantly lower with 8.66 ± 3.45 (*p* = 0.0024), indicating that on average the woody land was less fragmented than the crop. RDA was used to analyze the relationship between the 11 (4 landscape- and 7 local level) variables and the communities of parasitoids and drosophilids in Ticino. Results of separate RDAs are reported in Table 4. The full model for landscape level abiotic variables was not significant for the community of parasitoids, whereas it was marginally significant for the community composition of drosophilids (*p* = 0.097) explaining about 34% of the observed variation.

**Figure 1:**
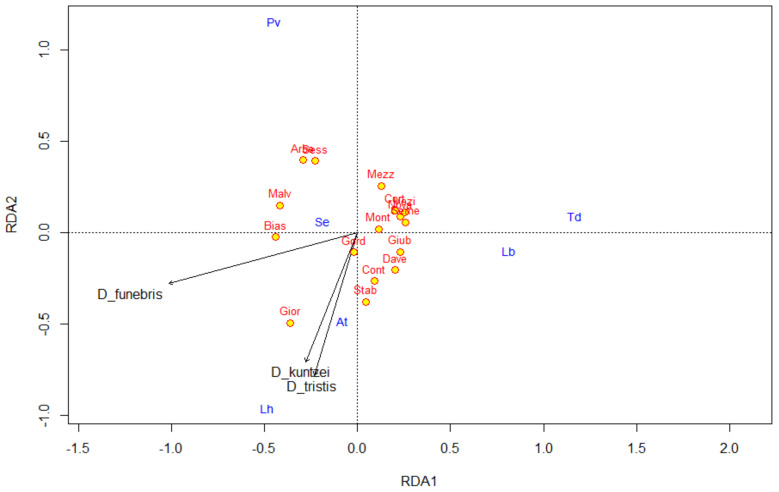
Redundancy analyses (RDA) ordination diagram for parasitoid species (in blue) and local scale biotic variables (arrows in black, abundance of three species of drosophilids) data collected in Ticino in 2017. The full model explained 52% of the total variance, the first two constrained axis explained 27% (RDA1) and 24% (RDA2). Red-bordered yellow dots are the sites (abbreviations in Table 1). At: *Asobara tabida*; Lb: *Leptopilina boulardi;* Lh: *Leptopilina heterotoma*; Pv: *Pachycrepoideus vindemmiae*; Se: *Spalangia erythromera*; Td: *Trichopria drosophilae.*

The full model for four local level variables (potential plant hosts of drosophilids) was not significant neither for the communities of parasitoids nor for drosophilids. The full model for three local level variables (potential drosophilid hosts of parasitoids), including three species of *Drosophila* after forward selection: *D. funebris*, *D. tristis* Fallen, 1823, and *D. kuntzei,* was highly significant (*p* = 0.001) and explained 52% of total variance. The variance explained by each species was 27% (*p* = 0.002), 13% (*p* = 0.02) and 12% (*p* = 0.02), respectively. The first constrained axis explained 27% (*p* = 0.004), while the second 24% (*p* = 0.008), with *D. funebris* negatively correlated with the first axis (−0.9486), whereas *D. tristis* and *D. kuntzei* were negatively correlated with the second axis (−0.7805 and −0.7041, respectively) (Figure 1).

#### 3.2.2. Influence of Habitat and Microhabitat Types on Community and on Single Species of Parasitoids

The community composition of the eight species of parasitoids was significantly affected by collecting period (*p* = 0.001), habitat (*p* = 0.003) and microhabitat (*p* = 0.001), as well as by the interaction between habitat and microhabitat (*p* = 0.002; Table 5). Analyses of the homogeneity of variance (PERMDISP) showed significant differences in community dispersion among sampling period (*p* = 0.006; Table 5) and specifically between sampling in May and the other months. Moreover, significant differences among habitat types (*p* = 0.02; Table 5), in particular between woody patches and crop, were found. The difference between microhabitat types (canopy versus ground) was not significant (*p* = 0.61; Table 5).

In short, from one assumption for PERMANOVA, i.e., homogeneous dispersion, fulfilled for Microhabitat, we can infer that the effect of microhabitat types on communities of parasitoids is ‘real’ and not an artifact of heterogeneous dispersions. For Period and Habitat variables, both PERMANOVA and PERMDISP were significant indicating that differences may be due to group (communities collected in different periods and habitat types) dispersions.

Pairwise comparisons showed that significantly more *L. heterotoma* (U = 16,897, *p* = 0.040) and *P. vindemmiae* (U = 14,457, *p* = 0.026) and marginally significantly more *S. erythromera* (U = 15,931, *p* = 0.055) emerged from traps placed in crop habitats compared to woody habitats. Emergence of the other species did not differ between the two habitat types. For all species microhabitat, i.e., the height of the Delta-trap above ground, significantly influenced the number of emerging parasitoids. While for *P. vindemmiae* significantly more individuals hatched from samples in the canopy (N = 264; U = 30,253, *p* < 0.001), all other species were found more often on the ground (*A. tabida*: U = 36,574, *p* = 0.002; *L. boulardi*: U = 22,558, *p* = 0.028; *L. heterotoma*: U = 38,781, *p* = 0.001; *T. drosophilae*: U = 36,815, *p* = 0.020, *T. modesta* (Ratzeburg, 1848): U = 3583, *p* < 0.001). *Spalangia erythromera* was exclusively found in traps on the ground.

#### 3.2.3. Host Preference of Parasitoid Species in Multi-Species Baited Delta-Traps

**Figure 2:**
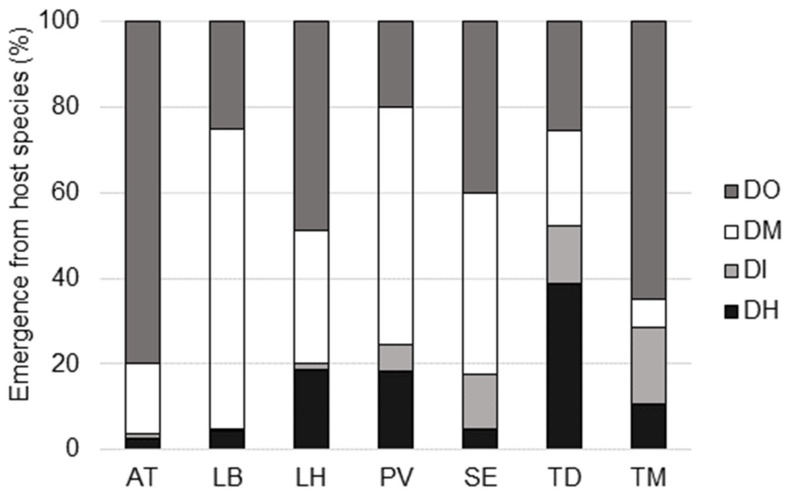
Host preference of parasitoid species in multi-species baited Delta-traps exposed in Ticino and Zurich in 2017. AT: *Asobara tabida*; LB: *Leptopilina boulardi*, LH: *Leptopilina heterotoma*; PV: *Pachycrepoideus vindemmiae*; SE: *Spalangia erythromera*; TD: *Trichopria drosophilae*; TM: *Trichopria modesta*; DO: *Drosophila subobscura*; DM: *Drosophila melanogaster*; DI: *Drosophila immigrans*; DH: *Drosophila hydei*.

For each species of parasitoids, the proportion of individuals emerging from different drosophilid species in multi-species baited Delta-traps is reported in Figure 2. The host species had a significant influence on the number of emerged *A. tabida* (X^2^ (3) = 25.154, *p* < 0.001), with significantly more offspring emerging from *D. melanogaster* and *D. subobscura* hosts compared to *D. hydei* and *D. immigrans* hosts. The emergence of *P. vindemmiae* (X^2^ (3) = 21.120, *p* < 0.001) and *T. modesta* (X^2^ (3) = 11.357, *p* < 0.010) was also significantly affected by host species. In the former, significantly more offspring emerged from *D. melanogaster*, in the latter from *D. subobscura* than from all other hosts. Similarly, *L. boulardi* emergence was significantly influenced by host species (X^2^(3) = 25.282, *p* < 0.001), with significantly more offspring emerging from *D. melanogaster* than from *D. immigrans* and *D. hydei*, and offspring from *D. subobscura* intermediate. In *L. heterotoma* highest numbers of offspring emerged from *D. subobscura*, followed by *D. melanogaster*, *D. hydei* and *D. immigrans* (X^2^ (3) = 107.418, *p* < 0.001). Host species had no significant effect on *T. drosophilae* and *S. erythromera*.

## 4. Discussion

The field sampling carried out in this study enabled us to characterize the communities of parasitoids and drosophilids in two different agricultural regions in Switzerland (Ticino and Zurich). The overall community assemblages of parasitoids differed between the agricultural regions in Ticino and Zurich according to the geographical distribution of the species recorded, with generalist species (e.g., *T. drosophilae* and *P. vindemmiae*) being widely distributed across both regions. The parasitoid communities of drosophilids were mainly influenced by the composition of their drosophilid hosts and by the microhabitat type (ground versus canopy). In particular, a high proportion of parasitoid community variation was explained by the presence of three species of Drosophilidae (*D. kuntzei*, *D. tristis*, and *D. funebris*) associated with two specialist parasitoids, *A. tabida* and *L. heterotoma*. All parasitoid species, except *P. vindemmiae*, were significantly associated with traps installed at ground level, indicating that microhabitat preference influences both parasitoid and drosophilid communities.

### 4.1. Species Composition in Zurich and Ticino

Eight species of hymenopteran parasitoids emerged from the traps baited with *Drosophila* infested fruit. In Ticino two species of *Leptopilina* were present with similar abundances at the regional level, with local differences in densities and distribution, while in Zurich only *L. heterotoma* was collected but representing the most abundant species. While sharing a similar ecological niche, these species differ in their geographic distribution with *L. boulardi* being mainly known from Mediterranean areas and currently expanding its range northwards [61]. Likewise, *T. drosophilae* was the only species in this genus collected in Ticino, whereas both species were collected in Zurich. *Trichopria modesta* appears to be distributed in Northern Europe [62]. While *T. drosophilae* is currently considered a candidate for the biological control of *D. suzukii* and thus its biology has been investigated into detail recently e.g., [18,36], little is known about *T. modesta*. However, our own experience suggests that *T. modesta* is less adapted to parasitize *D. suzukii* and has a markedly longer developmental time than *T. drosophilae* [63].

In Ticino, 27 species of Drosophilidae were recorded differing widely in abundance. All but five species (*D. melanogaster*, *D. simulans*, *C. amoena*, *Scaptomyza graminum*, and *S. pallida*) were caught in higher numbers in woody habitats (SNH and RH) than in the crop, even though some of them (e.g., *D. busckii*; *D. funebris*) are often referred to as domestic species, whereas others are already known as woodland species (e.g., *D. kuntzei*; *D. tristis*) ([64] and references therein). It is possible, however, that our traps differed in attractiveness compared to the background odor: a trap with odor similar to fermenting fruit (wine-vinegar) could be less attractive in an orchard at the time of fruit ripening compared to a woody habitat with fewer and smaller food resources for the flies.

### 4.2. Influence of Landscape Versus Local Variables on Community of Parasitoids and Drosophilids

Higher species diversity and population densities are generally considered to be positively related to non-crop habitats and patchiness in agricultural landscapes [6,7]. Landscape structure, such as habitat fragmentation, have been referred to as important factors shaping parasitoid assemblages [65]. Tscharntke and Brandl [66] reviewed the relationship of parasitoids’ traits with landscape structure and reported that the small body size favors dispersal of parasitoids in woody fragmented areas, while in agricultural area parasitoid richness and diversity dramatically decrease. Moreover, habitat fragmentation has also been shown to negatively affect specialized host-parasitoid associations (e.g., [67]). In our study, neither landscape structure nor composition at larger (500 m radius) or small (100 m) spatial scale had a significant effect on the community of parasitoids. The community of parasitoids observed in Ticino was mainly characterized by generalists, which are usually less influenced by habitat fragmentation [66]. However, species considered rather as specialists (e.g., *Asobara tabida*) were more strictly related to woody areas characterized by low habitat fragmentation in the studied region. Structure and composition of landscape also showed no effect on abundance and species composition of drosophilids, and only the overall model at broader landscape level (500 m) showed a certain trend toward significance (*p* value = 0.097), explaining 34% of the variation of the community. In a recent meta-analysis, Karp et al. [68] found that on average, 14%–20% in variation in pest control variables (e.g., pest abundance, natural enemy activity, yields, and many more) were explained by landscape composition, while a substantial amount of variation remained unaccounted. This suggests that the selection of variables to include in the model is crucial to increase the prediction capability of abundance and species composition. Furthermore, most of the studies investigating the effect of landscape composition on natural enemies and their hosts were conducted in annual crops where differences to semi-natural habitats in aspects such as microclimate and resource availability are sharper compared to permanent crops [68,69]. Permanent crops are less disturbed by management and may contain small pockets of spontaneous vegetation. Under these circumstances there might be sufficient resources, such as food or shelter, so that the effect of the surrounding landscape becomes negligible [11,68]. *Drosophila* species such as *D. subobscura* or *D. immigrans* are highly adaptable and can be found in forest and farmland alike [64] and the parasitoid *P. vindemmiae* has been described as inhabiting an extraordinarily broad range of habitats [70].

The large variation in the parasitoid community was explained by some of the *Drosophila* species inhabiting the investigated area in Ticino. We conclude that the community of parasitoids is to some extent driven by biotic interaction with some drosophilids previously reported as hosts in the literature. While the pupal parasitoids (e.g., *T. drosophilae* and *P. vindemmiae*) are rather generalist species regarding their host use, the larval parasitoids (*A. tabida*, *L. boulardi* and *L. heterotoma*) are more limited in their host choice and host suitability [71,72]. In our study, three drosophilid species, *D. funebris*, *D. tristis* and *D. kuntzei*, significantly accounted for a total of 52% variation in the parasitoid community. These species were previously recorded as hosts for *A. tabida* in the field [71]. In laboratory experiments, *D. funebris* was among the most preferred and suitable species for *A. tabida*, and *D. kuntzei* was the most suitable host among nine species tested for *L. heterotoma* [71,72]. Interestingly, only *D. tristis* uses fermenting fruit as main food source, whereas the other two species only occasionally occur on fruit and usually feed on fungi and decaying material [64,71]. Also, the parasitoids *A. tabida* and *L. heterotoma* were found to be associated with these latter food sources [73]. It is possible that in the studied regions, the interaction between parasitoids and drosophilids, associated with fungi, tree saps and decaying material, plays an important role early in the season when populations start to build up and fermenting fruit are still scarce. This idea is also supported by an earlier study revealing that *A. tabida* and *L. heterotoma* are among the earliest drosophilid parasitoids present as adults in spring [30]. In the present study, the three species of drosophilids were almost exclusively collected in woody area. The low fragmented woody land patches (SNH and RH) support a high proportion of specialized biotic associations and, compared to the agricultural land, seem to play a more important role in driving the overall parasitoid and drosophilids communities.

### 4.3. Influence of Habitat and Microhabitat Types on Community and on Single Species of Parasitoids

We showed that the microhabitat type (ground versus canopy) had a significant effect on the parasitoid assemblage. This result further supports the role of strict biotic associations between some species of parasitoids and drosophilid species associated with decomposition processes of plant litter. In our study, the significant effect of habitat type and sampling period on parasitoids was caused by the high variability of the data rather than the real effect of habitat type and season on parasitoids. No effect was visible for the ecotone, which then has to be considered to be a mere transition zone with no specific characteristics supporting parasitoid and drosophilid populations. The distribution of resources as well as the dispersal abilities of parasitoids determine their allocation within the landscape [8]. Besides hosts, food sources such as nectar or fruit saps and thermal conditions can play an important role here [5]. Furthermore, in our study it is possible that the structural complexity of the habitat (woody versus crop) as well as the attractiveness of the bait vs. the background can influence the trapping of species.

When inspected singularly, parasitoid species differed clearly in their microhabitat choice. All species, but *P. vindemmiae*, were collected in higher numbers at the ground compared to the canopy. *Pachycrepoideus vindemmiae* is a species with a particular broad host range, which includes Muscidae from birds’ nests and Tephritidae that oviposit into undamaged fruit [70]. On the contrary, native frugivorous drosophilids cannot lay their eggs into undamaged fruit but use damaged and fermenting fruit, which usually can be found on the ground, thus it seems adaptive that the more specialized parasitoids search primarily for hosts on the ground. The invasive *Drosophila suzukii* is able to infest ripening, undamaged fruit. Thus, larvae of this species will be found in larger amounts in the canopy, whereas at the time of pupation some fruit have already fallen from the plant and a proportion of individuals leaves the fruit to pupate on the ground [74].

### 4.4. Host Preference of Parasitoid Species in Multi-Species Baited Delta-Traps

The traps were baited with four frugivorous *Drosophila* species, belonging to different phylogenetic groups. When comparing numbers of emerged parasitoids from these samples, it has to be taken into account that number of specimens of each host provided might have been somewhat unequal due to differences in initial infestation and development success of certain species under field conditions. Thus, more *D. melanogaster* and less *D. hydei* and *D. immigrans* could have been available as hosts during the exposure. Also, the emergence from the samples represents a combination of preference and developmental success on the different species (apparent parasitism) while it does not account for any pre-imaginal mortality. Finally, since we introduced living, mobile hosts into the traps, it is possible that a few drosophilid individuals have moved from one sample into another. Despite the above considerations, remarkable significant differences in emergence between the parasitoid species, were detected in this study. Using traps exposed in the field, about 80% of *A. tabida* emerged from *D. subobscura*, and about 18% from *D. melanogaster*, which is in line with the finding that *D. subobscura* is the most preferred host species [71]. *Leptopilina boulardi* is considered to mainly parasitize *D. melanogaster* and *D. simulans*, whereas *L. heterotoma* is reported to have a broader host range [26]. However, in our samples, nearly 20% of *L. boulardi* emerged from *D. subobscura* as well as some from *D. hydei*, thus its host range may be broader than previously reported. While the larval parasitoids hardly parasitized *D. immigrans*, this species was used at least as a minor host by all pupal parasitoids. Most *T. modesta* emerged from *D. subobscura*, whereas in *T. drosophilae* no significant host preference was detected. While in our study we did not expose *D. suzukii* in the field due to non-native species regulations in Switzerland, in a laboratory study Boycheva Woltering et al. [75] found that *D. suzukii* was preferred among *D. melanogaster* and *D. immigrans* by *T. drosophilae*.

## 5. Conclusions

Our findings show that landscape composition and fragmentation, habitat type and seasonality did not affect the community composition of parasitoids. On the contrary, microhabitat and host type are the most important constrains, and these results are fully supported by the bionomic characteristics of the species recorded. The pupal parasitoids, which may use the exotic pest *D. suzukii*, have different preferences in terms of their microhabitat requirements. Due to its effective parasitization ability, *Trichopria drosophilae* has been preferred for augmentative control programs against *D. suzukii* [75]. However, the efficacy of the releases might be limited to pupae that are located on the ground. Thus, a combined release of *T. drosophilae* with *P. vindemmiae* could be essayed to target not only pupae on the ground but also those that remain in the canopy. So far, the only large-scale application of one of the species, *T. drosophilae* has been evaluated in a field study by Rossi Stacconi et al. [76]. While the collected pupal parasitoids might have some host preferences, they are generally broad in their host range. At the same time, our study also suggests that some of the larval parasitoids are more dependent on particular *Drosophila* species. Therefore, it is important to make sure that these species would not be severely affected by a large-scale application of pupal parasitoids.

## Figures and Tables

**Table 1:** List of the 24 localities investigated in 2017 in Switzerland. Locality, coordinates and altitude are reported for both Ticino and Zurich region, and landscape type for Ticino.

Canton ^1^	Locality	Site Code	Landscape Type ^2^	Lat. [N]/Lon. [E] ^3^	Elevation [m a.s.l.]
TI	Vezia	1-Vezi	Homo	46°01′16″/8°55′55″	334.5
TI	Giornico	2-Gior	Homo	46°23′48″/8°52′36″	377.3
TI	Contone	3-Cont	Hete	46°08′54″/8°55′47″	211.4
TI	Arbedo	4-Arbe	Hete	46°13′11″/9°03′13″	264.0
TI	Davesco	5-Dave	Hete	46°01′48″/8°58′30″	377.9
TI	Corteglia	6-Cort	Homo	45°51′51″/8°59′36″	426.4
TI	Mezzana	7-Mezz	Hete	45°51′08″/8°59′58″	327.1
TI	Stabio	8-Stab	Homo	45°51′13″/8°55′36″	409.4
TI	Gordola	9-Gord	Homo	46°10′53″/8°52′12″	216.1
TI	Sementina	10-Seme	Homo	46°10′52″/8°58′27″	374.9
TI	Malvaglia	11-Malv	Homo	46°24′34″/8°59′01″	429.9
TI	Novazzano	12-Nova	Hete	45°50′41″/8°57′57″	378.0
TI	Sessa	13-Sess	Homo	46°00′30″/8°49′46″	527.3
TI	Monteggio	14-Mont	Hete	45°59′44″/8°48′51″	408.8
TI	Biasca	15-Bias	Hete	46°20′47″/8°58′10″	283.5
TI	Giubiasco	16-Giub	Hete	46°09′49″/8°58′48″	211.4
ZH	Reckenholz	17-Reck	-	47°25′45″/8°31′51″	442
ZH	Waidhof	18-Waid	-	47°25′23″/8/31′36″	457
ZH	Seebach	19-Seeb	-	47°25′29″/8°31′60″	436
ZH	Rieder	20-Ried	-	47°25′50″/8°31′40″	447
ZH	Rümlang	21-Ruml	-	47°26′10″/8°32′15″	463
ZH	Bahn	22-Bahn	-	47°25′08″/8°30′59″	456
ZH	Glaubten	23-Glau	-	47°24′35″/8°31′18″	495
ZH	Buchegg	24-Buch	-	47°24′09″/8°31′49″	478

^1^ TI: Ticino (Southern Switzerland); ZH: Zurich (Northern Switzerland). ^2^ Homo: homogeneous; Hete: heterogeneous. ^3^ Lat.: latitude; Lon.: longitude.

**Table 2:** Multiscale approach used to analyse the communities of parasitoids and their potential drosophilid hosts. DH: *Drosophila hydei*; DI: *D. immigrans*; DM: *D. melanogaster*; DO: *D. subobscura*. For each spatial scale investigated, the list of the response and explanatory variables used in the analyses is reported.

Spatial Scale	Response Variables	Explanatory Variables	Analyses ^1^
Landscape (500 m radius)	Parasitoid and drosophilid communities	% crop cover % woody cover Crop patch complexity Woody patch complexity	Community level
Local (100 m radius)	Parasitoid and drosophilid communities	% vineyards % berries Presence wild-berries Presence wild trees Drosophilid species	Community level
Habitat (<50 m radius)	Parasitoid communities and single species of parasitoids	Habitat type (crop, woody, ecotone) period	Community and Single species level
Microhabitat (<0.2 m radius)	Parasitoid communities and single species of parasitoids	Microhabitat type (ground, canopy) period	Community and Single species level
Within Delta-trap (Host preference)	Single species of parasitoids	Host species (DH, DI, DM, DO)	Single species level

^1^ Community level analyses are performed using a multivariate approach: Redundancy analyses (RDA) and distance-based permutational multivariate analysis of variance (PERMANOVA); Single species-level analyses are performed using a pairwise comparison approach: non-parametric Man-Whitney U tests.

**Table 3:** Parasitoid species emerged from Delta-traps baited with four Drosophilidae species (*Drosophila hydei*, *D. immigrans*, *D. melanogaster*, and *D. subobscura*) in Ticino and Zurich in 2017.

Species	Host Stage Parasitized	Ticino			Zurich		
		# Traps ^1^	# Ind. ^2^	# Sites ^3^	# Traps	# Ind.	# Sites
**Braconidae**							
*Asobara tabida*	larva	8	34	4	11	119	7
**Figitidae**							
*Leptopilina boulardi*	larva	17	649	8	-	-	-
*Leptopilina heterotoma*	larva	16	426	11	83	5316	8
**Diapriidae**							
*Trichopria drosophilae*	pupa	44	926	13	1	24	1
*Trichopria modesta*	pupa	-	-	-	21	204	8
**Pteromalidae**							
*Pachycrepoideus vindemmiae*	pupa	27	646	13	16	289	8
*Spalangia erythromera*	pupa	3	13	3	5	27	4
*Vrestovia brevior*	pupa	1	4	1	-	-	-

^1^ number of traps containing the species; ^2^ ind.: number of individuals collected; ^3^ number of sites where the species has been collected.

**Table 4:** Results of separate RDA analyses performed to test for effect of landscape- and local level variables on the community composition of both parasitoids and drosophilids in Ticino in 2017.

Environmental Variables	Parasitoids		Drosophilids	
	R2adj ^a^	*p* Value ^b^	R2adj ^a^	*p* Value ^b^
**Landscape level**				
crop_500	-	ns	-	ns
woody_500	-	ns	-	ns
PD_crop_500	-	ns	-	ns
PD_woody_500	-	ns	-	ns
Total	23%	ns	34%	.
**Local level (potential plant hosts of drosophilids)**				
vineyard_100	-	ns	-	ns
berries_100	-	ns	-	ns
berries_wild	-	ns	-	ns
tree_wild	-	ns	-	ns
Total	20%	ns	27%	ns
**Local level (potential drosophilid hosts of parasitoids)**				
D_funebris	27%	**	-	-
D_tristis	13%	*	-	-
D_kuntzei	12%	*	-	-
Total	52%	***	-	-

^a^: percentage of explained variation. ^b^: *** (*p* ≤ 0.001); ** (*p* ≤ 0.01); * (*p* ≤ 0.05); ‘.’ (*p* ≤ 0.1); ns = not significant.

**Table 5:** PERMANOVA and PERMDISP analysis of the effect of collecting Period (5 periods), Habitat type (crop, ecotone and woody), and Microhabitat (canopy and ground) on community composition of parasitoids associated with four different species of Drosophilidae collected in Ticino and Zurich in 2017. Number of permutations: 999. The factor site was included as random factor. Df: degrees of freedom; SS: sum of squares; MS: mean squares; Pseudo F: F value by permutation; *p* (perm): *p* value by permutation; EV: variance explained.

Source of Variation	Df	SS	MS	Pseudo F	*p* (perm) ^a^	EV (%)
**PERMANOVA**						
Period	4	7.74	1.93	6.38	0.001	11.1
Habitat	2	3.02	1.51	4.99	0.003	4.3
Microhabitat	1	3.56	3.56	11.73	0.001	5.1
Period × Habitat	6	2.91	4.50	1.60	ns	4.2
Period × Microhabitat	4	1.39	0.35	1.15	ns	2.0
Habitat × Microhabitat	2	1.70	0.85	2.80	0.002	2.4
Period × Habitat × Microhabitat	6	2.24	0.37	1.23	ns	3.2
Residuals	156	47.28	0.30	-	-	67.7
Total	181	69.83	-	-	-	100
**PERMDISP**						
Period	4	-	-	3.74	0.006	-
Total	177	-	-	-	-	-
May–Jun	-	-	-	-	0.03	-
May–Aug	-	-	-	-	0.06	-
May–Sep	-	-	-	-	0.001	-
May–Oct	-	-	-	-	0.07	-
Jun–Aug	-	-	-	-	ns	-
Jun–Sep	-	-	-	-	ns	-
Jun–Oct	-	-	-	-	ns	-
Aug–Sep	-	-	-	-	ns	-
Aug–Oct	-	-	-	-	ns	-
Sep–Oct	-	-	-	-	ns	-
Habitat	2	-	-	3.96	0.02	-
Total	179	-	-	-	-	-
ecotone-crop	-	-	-	-	ns	-
woody-crop	-	-	-	-	0.02	-
woody-ecotone	-	-	-	-	ns	-
Microhabitat	1	-	-	0.25	ns	-
Total	180	-	-	-	-	-

^a^: boldface indicates statistical significance at *p* < 0.05; ns = not significant.

## Supplementary Materials

The following are available online at https://www.mdpi.com/2075-4450/11/6/334/s1, Figure S1: Example of a handmade modified Delta-trap (13 × 20 × 7 cm W × L × H) baited with larvae and pupae of four native drosophilid species (*Drosophila hydei*- DH, *D. immigrans*- DI, *D. melanogaster*- DM and *D. subobscura*- DO) allocated in four plastic cups (dressing dishes, PS round 50 mL Ø 6,7 cm, 2,7 cm clear, PAPSTAR). The cups were filled with ripe seasonal fruit and allocated inside a support covered by a rounded roof; Figure S2: Relative abundance and distribution of parasitoid species collected in 16 localities in Ticino in 2017. AT: *Asobara tabida*; LB: *Leptopilina boulardi*; LH: *Leptopilina heterotoma*; PV: *Pachycrepoideus vindemmiae*; SE: *Spalangia erythromera*; TD: *Trichopria drosophilae*; VF: *Vrestovia brevior*; Table S1: List of potential host plants of Drosophilidae recorded in a buffer of 100-m radius surrounding Gasser-Becherfalle traps in Ticino in 2017 and the assigned code of preference; Table S2: Abundance of Drosophilidae sampled in 16 sites in Ticino. Specimens were collected using two Gasser-Becherfalle traps (Organic fruit fly traps for *Drosophila suzukii*, Riga AG, Switzerland) in each site and five one-week sampling period from May to October 2017. For each species, the proportion of specimens collected in woody habitat is also reported (% Ind. Woody). Abbreviations of sites in Table 1.

## References

[1] Quicke D.L. (1997). Parasitic Wasps.

[2] Clarke C.W., Calatayud P.A., Sforza R.F., Ndemah R.N., Nyamukondiwa C. (2019). Parasitoids’ Ecology and Evolution. Front. Ecol. Evol..

[3] Thierry M., Hrček J., Lewis O.T. (2019). Mechanisms structuring host–parasitoid networks in a global warming context: A review. Ecol. Entomol..

[4] Fleury F., Ris N., Allemand R., Fouillet P., Carton Y., Boulétreau M., Capy P., Gibert P., Boussy I. (2004). Ecological and genetic interactions in Drosophila-parasitoids communities: A case study with D. melanogaster, D. simulans and their common Leptopilina parasitoids in south-eastern France. Drosophila Melanogaster, Drosophila Simulans: So Similar, so Different.

[5] Gillespie M.A., Gurr G.M., Wratten S.D. (2016). Beyond nectar provision: The other resource requirements of parasitoid biological control agents. Entomol. Exp. Appl..

[6] Bianchi F.J., Booij C.J.H., Tscharntke T. (2006). Sustainable pest regulation in agricultural landscapes: A review on landscape composition, biodiversity and natural pest control. Proc. R. Soc. B Biol. Sci..

[7] Veres A., Petit S., Conord C., Lavigne C. (2013). Does landscape composition affect pest abundance and their control by natural enemies? A review. Agric. Ecosyst. Environ..

[8] Rusch A., Bommarco R., Ekbom B. (2017). Conservation biological control in agricultural landscapes. Adv. Bot. Res..

[9] Lavandero B., Wratten S., Shishehbor P., Worner S. (2005). Enhancing the effectiveness of the parasitoid Diadegma semiclausum (Helen): Movement after use of nectar in the field. Biol. Control.

[10] Tylianakis J.M., Didham R.K., Wratten S.D. (2004). Improved fitness of aphid parasitoids receiving resource subsidies. Ecology.

[11] Tscharntke T., Karp D.S., Chaplin-Kramer R., Batáry P., DeClerck F., Gratton C., Hunt L., Ives A., Jonsson M., Larsen A. (2016). When natural habitat fails to enhance biological pest control—Five hypotheses. Biol. Conserv..

[12] De Ros G., Anfora G., Grassi A., Ioriatti C. (2013). The potential economic impact of Drosophila suzukii on small fruits production in Trentino (Italy). IOBC-WPRS Bull..

[13] Mazzi D., Bravin E., Meraner M., Finger R., Kuske S. (2017). Economic impact of the introduction and establishment of Drosophila suzukii on sweet cherry production in Switzerland. Insects.

[14] Asplen M.K., Anfora G., Biondi A., Choi D.S., Chu D., Daane K.M., Gibert P., Gutierrez A.P., Hoelmer K.A., Hutchison W.D. (2015). Invasion biology of spotted wing Drosophila (Drosophila suzukii): A global perspective and future priorities. J. Pest Sci..

[15] Deprá M., Poppe J.L., Schmitz H.J., De Toni D.C., Valente V.L. (2014). The first records of the invasive pest Drosophila suzukii in the South American continent. J. Pest Sci..

[16] Kenis M., Tonina L., Eschen R., van der Sluis B., Sancassani M., Mori N., Haye T., Helsen H. (2016). Non-crop plants used as hosts by Drosophila suzukii in Europe. J. Pest Sci..

[17] Poyet M., Le Roux V., Gibert P., Meirland A., Prevost G., Eslin P., Chabrerie O. (2015). The wide potential trophic niche of the Asiatic fruit fly Drosophila suzukii: The key of its invasion success in temperate Europe?. PLoS ONE.

[18] Wolf S., Boycheva-Woltering S., Romeis J., Collatz J. (2020). Trichopria drosophilae parasitizes Drosophila suzukii in seven common non-crop fruits. J. Pest Sci..

[19] Tait G., Grassi A., Pfab F., Crava C.M., Dalton D.T., Magarey R., Ometto L., Vezzulli S., Rossi-Stacconi M.V., Gottardello A. (2018). Large-scale spatial dynamics of Drosophila suzukii in Trentino, Italy. J. Pest Sci..

[20] Bruck D.J., Bolda M., Tanigoshi L., Klick J., Kleiber J., DeFrancesco J., Gerdeman B., Spitler H. (2011). Laboratory and field comparisons of insecticides to reduce infestation of Drosophila suzukii in berry crops. Pest Manag. Sci..

[21] Pelton E., Gratton C., Isaacs R., Van Timmeren S., Blanton A., Guédot C. (2016). Earlier activity of Drosophila suzukii in high woodland landscapes but relative abundance is unaffected. J. Pest Sci..

[22] Cahenzli F., Bühlmann I., Daniel C., Fahrentrapp J. (2018). The distance between forests and crops affects the abundance of Drosophila suzukii during fruit ripening, but not during harvest. Environ. Entomol..

[23] Leach H., Moses J., Hanson E., Fanning P., Isaacs R. (2018). Rapid harvest schedules and fruit removal as non-chemical approaches for managing spotted wing Drosophila. J. Pest Sci..

[24] Haro-Barchin E., Scheper J., Ganuza C., De Groot G.A., Colombari F., van Kats R., Kleijn D. (2018). Landscape-scale forest cover increases the abundance of Drosophila suzukii and parasitoid wasps. Basic Appl. Ecol..

[25] Santoiemma G., Mori N., Tonina L., Marini L. (2018). Semi-natural habitats boost Drosophila suzukii populations and crop damage in sweet cherry. Agric. Ecosyst. Environ..

[26] Carton Y., Boulétreau M., van Alphen J.J.M., van Lenteren J.C., Ashburner M., Carson H.L., Thompson J.N.J. (1986). The Drosophila parasitic wasps. The Genetics and Biology of Drosophila.

[27] Miller B., Anfora G., Buffington M., Daane K.M., Dalton D.T., Hoelmer K.M., Rossi Stacconi M.V., Grassi A., Ioriatti C., Loni A. (2015). Seasonal occurrence of resident parasitoids associated with Drosophila suzukii in two small fruit production regions of Italy and the USA. Bull. Insectol..

[28] Mazzetto F., Marchetti E., Amiresmaeili N., Sacco D., Francati S., Jucker C., Dindo M.L., Lupi D., Tavella L. (2016). Drosophila parasitoids in northern Italy and their potential to attack the exotic pest Drosophila suzukii. J. Pest Sci..

[29] Gabarra R., Riudavets J., Rodríguez G.A., Pujade-Villar J., Arnó J. (2015). Prospects for the biological control of Drosophila suzukii. BioControl.

[30] Knoll V., Ellenbroek T., Romeis J., Collatz J. (2017). Seasonal and regional presence of hymenopteran parasitoids of Drosophila in Switzerland and their ability to parasitize the invasive Drosophila suzukii. Sci. Rep..

[31] Kremmer L., Thaon M., Borowiec N., David J., Poirié M., Gatti J.L., Ris N. (2017). Field monitoring of Drosophila suzukii and associated communities in south eastern France as a pre-requisite for classical biological control. Insects.

[32] Chabert S., Allemand R., Poyet M., Eslin P., Gibert P. (2012). Ability of European parasitoids (Hymenoptera) to control a new invasive Asiatic pest, Drosophila suzukii. Biol. Control.

[33] Iacovone A., Ris N., Poirié M., Gatti J.-L. (2018). Time-course analysis of Drosophila suzukii interaction with endoparasitoid wasps evidences a delayed encapsulation response compared to D. melanogaster. PLoS ONE.

[34] Rossi Stacconi M.V., Grassi A., Dalton D.T., Miller B., Ouantar M., Loni A., Ioriatti C., Walton V.M., Anfora G. (2013). First field records of Pachycrepoideus vindemiae as a parasitoid of Drosophila suzukii in European and Oregon small fruit production areas. Entomologia.

[35] Rossi Stacconi M.V., Panel A., Baser N., Ioriatti C., Pantezzi T., Anfora G. (2017). Comparative life history traits of indigenous Italian parasitoids of Drosophila suzukii and their effectiveness at different temperatures. Biol. Control.

[36] Amiresmaeili N., Jucker C., Savoldelli S., Lupi D. (2018). Understanding Trichopria drosophilae performance in laboratory conditions. Bull. Insectol..

[37] Wang X.G., Serrato M.A., Son Y., Walton V.M., Hogg B.N., Daane K.M. (2018). Thermal performance of two indigenous pupal parasitoids attacking the invasive Drosophila suzukii (Diptera: Drosophilidae). Environ. Entomol..

[38] Giorgini M., Wang X.G., Wang Y., Chen F.S., Hougardy E., Zhang H.M., Chen Z.Q., Chen H.Y., Liu C.X., Cascone P. (2019). Exploration for native parasitoids of Drosophila suzukii in China reveals a diversity of parasitoid species and narrow host range of the dominant parasitoid. J. Pest Sci..

[39] Lue C.H., Borowy D., Buffington M.L., Leips J. (2018). Geographic and seasonal variation in species diversity and community composition of frugivorous Drosophila (Diptera: Drosophilidae) and their Leptopilina (Hymenoptera: Figitidae) parasitoids. Environ. Entomol..

[40] Spinedi F., Isotta F. (2004). Il clima del Ticino. Dati Statistiche E Società.

[41] Weber M., Sorg L., Flury C. (2014). Landwirtschaft und Landschaft im Kanton Zürich. Handlungsbedarf für die Kantonale Politik.

[42] DWD Deutscher Wetterdiens. https://www.dwd.de/DE/leistungen/klimadatenwelt/europa/rs/schweiz/schweiz_node.html.

[43] McGarigal K., Cushman S.A., Neel M.C., Ene E. (2002). FRAGSTATS: Spatial Pattern Analysis Program for Categorical Maps. Documentation of the Computer Software Program.

[44] Lee J.C., Dreves A.J., Cave A.M., Kawai S., Isaacs R., Miller J.C., van Timmeren S., Bruck D.J. (2015). Infestation of wild and ornamental noncrop fruits by Drosophila suzukii (Diptera: Drosophilidae). Ann. Entomol. Soc. Am..

[45] Perkins R.C.L., Sharp D. (1910). Hymenoptera (supplement). Fauna Hawaiiensis.

[46] Nixon G.E.J., Fitton M.G. (1980). Diapriidae (Diapriinae) Hymenoptera, Proctotrupoidea. Handbooks for the Identification of British Insects.

[47] Graham M.W.R.D.V. (1969). The Pteromalidae of north-western Europe (Hymenoptera-Chalcidoidea). Bull. Br. Mus. Nat. Hist. Entomol..

[48] Forshage M., Nordlander G. (2008). Identification key to European genera of Eucoilinae (Hymenoptera, Cynipoidea, Figitidae). Insect Syst. Evol..

[49] Lue C.H., Driskell A.C., Leips J., Buffington M.L. (2016). Review of the genus Leptopilina (Hymenoptera, Cynipoidea, Figitidae, Eucoilinae) from the Eastern United States, including three newly described species. J. Hymenopt. Res..

[50] Nordlander G. (1980). Revision of the genus Leptopilina Förster, 1869, with notes on the status of some other genera (Hymenoptera, Cynipoidea: Eucoitidae). Insect Syst. Evol..

[51] Bächli G., Viljoen F., Escher S.A., Saura A. (2005). The Drosophilidae (Diptera) of Fennoscandia and Denmark.

[52] Dray S., Blanchet F.G., Legendre P. (2013). Packfor: Forward Selection with Permutation (Canoco p. 46). http://R-Forge.R-project.org/projects/sedar.

[53] Legendre P., Gallagher E.D. (2001). Ecologically meaningful transformations for ordination of species data. Oecologia.

[54] Anderson M.J. (2001). A new method for non-parametric multivariate analysis of variance. Austral. Ecol..

[55] Bray J.R., Curti J.T. (1957). An ordination of upland forest communities of southern Wisconsin. Ecol. Monogr..

[56] Anderson M., Ter Braak C. (2003). Permutation tests for multi-factorial analysis of variance. J. Stat. Comput. Simul..

[57] Anderson M.J. (2006). Distance-based tests for homogeneity of multivariate dispersions. Biometrics.

[58] Anderson M.J., Ellingsen K.E., McArdle B.H. (2006). Multivariate dispersion as a measure of beta diversity. Ecol. Lett..

[59] Benjamini Y., Hochberg Y. (1995). Controlling the false discovery rate: A practical and powerful approach to multiple testing. J. R. Stat. Soc..

[60] R Core Team (2014). R: A Language and Environment for Statistical Computing.

[61] Patot S., Martinez J., Allemand R., Gandon S., Varaldi J., Fleury F. (2010). Prevalence of a virus inducing behavioural manipulation near species range border. Mol. Ecol..

[62] De Jong Y., Verbeek M., Michelsen V., de Place Bjørn P., Los W., Steeman F., Bailly N., Basire C., Chylarecki P., Stloukal E. (2014). Fauna Europaea—All European animal species on the web. Biodivers. Data J..

[63] Collatz J. (2019). Personal communication.

[64] Shorrocks B. (1977). An ecological classification of European Drosophila species. Oecologia.

[65] Cooke B., Roland J. (2000). Spatial analysis of large-scale patterns of forest tent caterpillar outbreaks. Ecoscience.

[66] Tscharntke T., Brandl R. (2004). Plant-insect interactions in fragmented landscapes. Annu. Rev. Entomol..

[67] Anderson R.M., Dallar N.M., Pirtel N.L., Connors C.J., Mickley J., Bagchi R., Singer M.S. (2019). Bottom-Up and top-down effects of forest fragmentation differ between dietary generalist and specialist caterpillars. Front. Ecol. Evol..

[68] Karp D.S., Chaplin-Kramer R., Meehan T.D., Martin E.A., DeClerck F., Grab H., Gratton C., Hunt L., Larsen A.E., Martínez-Salinas A. (2018). Crop pests and predators exhibit inconsistent responses to surrounding landscape composition. Proc. Natl. Acad. Sci. USA.

[69] Boccaccio L., Petacchi R. (2009). Landscape effects on the complex of Bactrocera oleae parasitoids and implications for conservation biological control. BioControl.

[70] Peters R.S. (2009). New habitat and host records and notes on the life history of Pachycrepoideus vindemmiae (Rondani, 1875). Mitt. Hamb. Zool. Mus. Inst..

[71] Van Alphen J.J.M., Janssen A.R.M. (1982). Host selection by Asobara tabida Nees (Braconidae; Alysiinae) a larval parasitoid of fruit inhabiting Drosophila species, 2: Host species selection. Neth. J. Zool..

[72] Janssen A. (1989). Optimal host selection by Drosophila parasitoids in the field. Funct. Ecol..

[73] Janssen A., Driessen G., De Haan M., Roodbol N. (1988). The impact of parasitoids on natural populations of temperate woodland Drosophila. Neth. J. Zool..

[74] Woltz J.M., Lee J.C. (2017). Pupation behavior and larval and pupal biocontrol of Drosophila suzukii in the field. Biol. Control.

[75] Boycheva Woltering S., Romeis J., Collatz J. (2019). Influence of the rearing host on biological parameters of Trichopria drosophilae, a potential biological control agent of Drosophila suzukii. Insects.

[76] Rossi Stacconi M.V., Grassi A., Ioriatti C., Anfora G. (2019). Augmentative releases of Trichopria drosophilae for the suppression of early season Drosophila suzukii populations. BioControl.

