# Supplementary material for: Multiscale Determinants Drive Parasitization of Drosophilidae by Hymenopteran Parasitoids in Agricultural Landscapes
Source: Insects. 2020 May 30;11(6):334. doi: 10.3390/insects11060334 (PMC7348750; doi:10.3390/insects11060334)
Supplement: Supplementary file 1 [file insects-11-00334-s001.zip › insects-806104-supplementary/Figure_S1.docx]

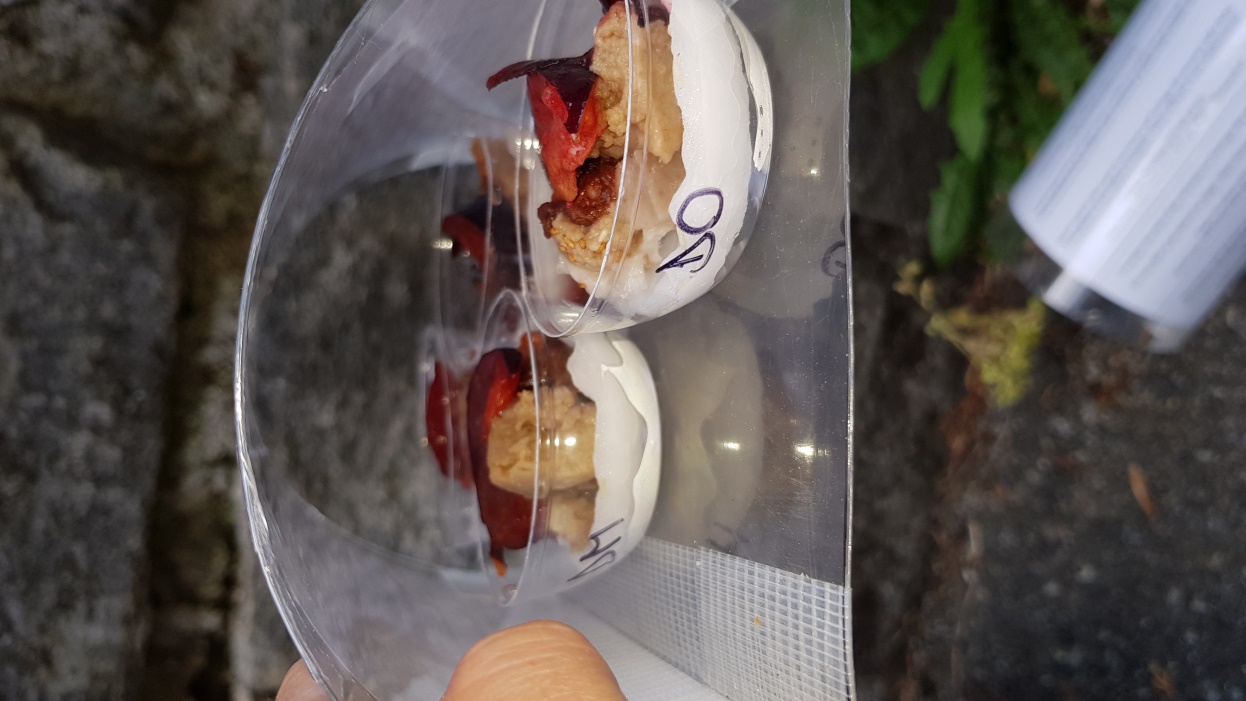


**Figure S1.** Example of a handmade modified Delta-trap (13 × 20 × 7 cm W × L × H) baited with larvae and pupae of 4 native drosophilid species (*Drosophila hydei*- DH, *D. immigrans*- DI, *D. melanogaster*- DM and *D. subobscura*- DO) allocated in 4 plastic cups (dressing dishes, PS round 50 ml Ø 6,7 cm, 2,7 cm clear, PAPSTAR). The cups were filled with ripe seasonal fruits and allocated inside a support covered by a rounded roof.

| 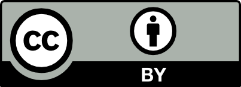 | © 2019 by the authors. Submitted for possible open access publication under the terms and conditions of the Creative Commons Attribution (CC BY) license (http://creativecommons.org/licenses/by/4.0/). |
| --- | --- |
