# Supplementary material for: Multiscale Determinants Drive Parasitization of Drosophilidae by Hymenopteran Parasitoids in Agricultural Landscapes
Source: Insects. 2020 May 30;11(6):334. doi: 10.3390/insects11060334 (PMC7348750; doi:10.3390/insects11060334)
Supplement: Supplementary file 1 [file insects-11-00334-s001.zip › insects-806104-supplementary/Figure_S2.docx]

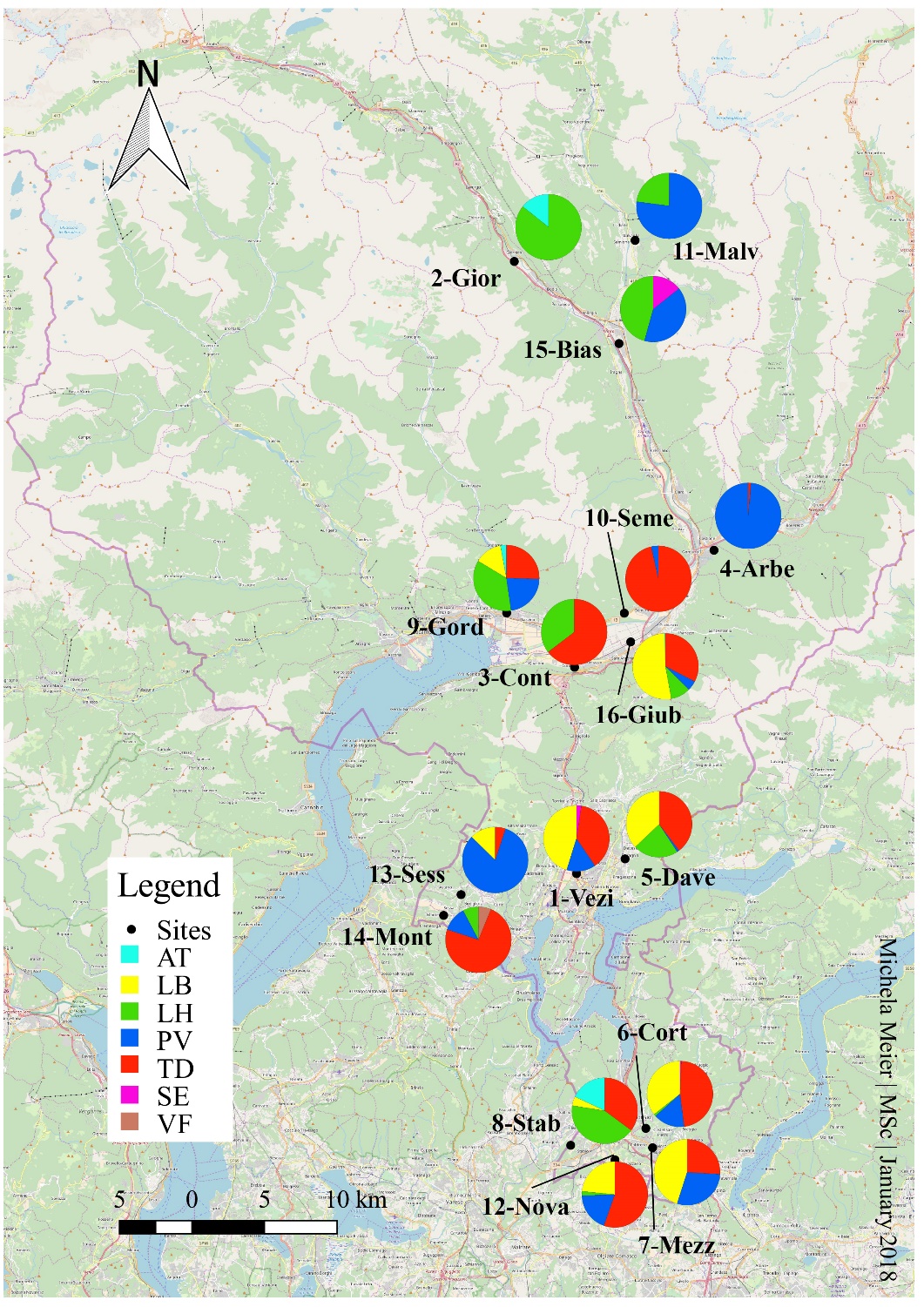


**Figure S2.** Relative abundance and distribution of parasitoid species collected in 16 localities in Ticino in 2017. AT: *Asobara tabida*; LB: *Leptopilina boulardi*; LH: *Leptopilina heterotoma*; PV: *Pachycrepoideus vindemmiae*; SE: *Spalangia erythromera*; TD: *Trichopria drosophilae*; VF: *Vrestovia brevior*. 1-Vezi: Vezia; 2-Gior: Giornico; 3- Cont: Contone; 4-Arbe: Arbedo; 5-Dave: Davesco; 6-Cort: Corteglia; 7-Mezz: Mezzana; 8-Stab: Stabio; 9-Gord: Gordola; 10-Seme: Sementina; 11-Malv: Malvaglia; 12-Nova: Novazzano; 13-Sess: Sessa; 14-Mont: Monteggio; 15-Bias: Biasca; 16-Giub: Giubiasco.

| 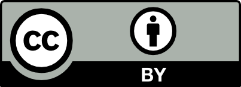 | © 2019 by the authors. Submitted for possible open access publication under the terms and conditions of the Creative Commons Attribution (CC BY) license (http://creativecommons.org/licenses/by/4.0/). |
| --- | --- |
