# Supplementary material for: Multiscale Determinants Drive Parasitization of Drosophilidae by Hymenopteran Parasitoids in Agricultural Landscapes
Source: Insects. 2020 May 30;11(6):334. doi: 10.3390/insects11060334 (PMC7348750; doi:10.3390/insects11060334)
Supplement: Supplementary file 1 [file insects-11-00334-s001.zip › insects-806104-supplementary/Table S1.docx]

**Table S1.** List of potential host plants of Drosophilidae recorded in a buffer of 100-m radius surrounding Gasser-Becherfalle traps in Ticino in 2017 and the assigned code of preference.

| **Potential host plant** | **Code^1^** | **Site** |
| --- | --- | --- |
| *Rubus fruticosus* | 2 | 1-Vezi,2-Gior,3-Cont, 5-Dave, 6-Cort,7-Mezz,8-Stab,9-Gord,10-Seme,11-Malv,12-Nova,14-Mont,15-Bias |
| *Prunus spp.* | 1 | 5-Dave |
| *Ficus carica* | 2 | 5-Dave,6-Cort,9-Gord,10-Seme,13-Sess, |
| *Sambucus sp.* | 1 | 13-Sess,14-Mont |
| *Phytolacca americana* | 2 | 1-Vezi, ,3-Cont,6-Cort,8-Stab, 9-Gord, 14-Mont,15-Bias |
| *Prunus avium* (wild cherry) | 1 | 3-Cont,11-Malv,12-Nova, 14-Mont |

^1^ ordinal code defined after Figure 3 in [16] and also corroborated in [17,44].

1: suitable plant host; 2: preferred plant host.

| 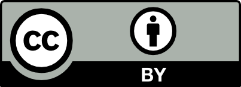 | © 2019 by the authors. Submitted for possible open access publication under the terms and conditions of the Creative Commons Attribution (CC BY) license (http://creativecommons.org/licenses/by/4.0/). |
| --- | --- |
